# Non-pharmacological interventions in dementia care: what is being implemented

**DOI:** 10.1192/bjb.2025.10120

**Published:** 2026-04

**Authors:** Sabrina D. Ross, Nadja Ziegert, Francisca S. Rodriguez

**Affiliations:** German Center for Neurodegenerative Diseases (DZNE), Greifswald, Germany

**Keywords:** Dementias/neurodegenerative diseases, out-patient treatment, psychosocial interventions, old age psychiatry, psychiatric nursing

## Abstract

**Aims and method:**

Non-pharmacological interventions (NPIs) are recognised for their potential in treating dementia symptoms. However, little is known about the extent of their use. In this study, we conducted structured interviews with people with dementia and their family caregivers (*n* = 50), professional caregivers (*n* = 42) and dementia care coordinators (*n* = 42) on the use of 14 NPIs.

**Results:**

Cognitive stimulation/training, physical activity and occupational therapy were implemented by most participants, whereas neurofeedback, drama therapy and phototherapy were rarely used. Most NPIs were carried out weekly. People with dementia and their caregivers reported using significantly fewer NPIs than other participants (*P* < 0.001). Participants perceived effects for, on average, 90.3% (s.d. = 31.3%) of the NPIs that they used.

**Clinical implications:**

Providing targeted support and funding might help to increase the use of NPIs by family caregivers as well as in institutional care settings.

With an ageing population,^1^ the number of people with dementia is increasing,^2^ placing an increasing burden on caregivers, the healthcare system and society as a whole.^3,4^ Beyond memory loss, dementia includes behavioural and psychological symptoms (BPSD) that affect cognitive function, daily activities and the quality of life of those with dementia^5^ and therefore increase caregiver burden.^6^ Although pharmacological solutions can provide some relief, non-pharmacological interventions (NPIs) have also been shown to alleviate dementia symptoms (e.g. cognitive performance, emotional well-being, functional abilities^7,8^). NPIs include a wide range of approaches, such as cognitive stimulation,^9^ aromatherapy,^10^ animal-assisted therapy^11^ and music therapy.^12^ National and international guidelines, e.g. by the World Health Organization,^13^ recommend implementing NPIs in dementia care. Nonetheless, there is a lack of knowledge on the extent to which they are actually utilised in dementia care. Despite beneficial effects reported in systematic reviews^14,15^ and by stakeholders,^16,17^ barriers may hinder utilisation.^16,18^

To get an overview of the current situation, we asked people with dementia and those involved in their care about their experiences of NPI use in dementia care. We specifically assessed the types of intervention, their frequency, funding and perceived effects.

## Method

### Sample

As part of a study of interventions in dementia care from the perspective of different stakeholders, this paper focuses specifically on NPIs. Participants were recruited nationwide by telephone or email via strategic sampling to minimise selection bias. Initially, physicians, therapists and nursing homes were contacted. Subsequently, we systematically contacted nursing care networks, dementia networks, support groups, geriatric care working groups, professional associations and organisers of nursing training courses across all the federal states of Germany. Inclusion criteria were being at least 18 years old, actively involved in dementia care, capable of giving consent (i.e. not currently experiencing delirium), and having sufficient visual and hearing abilities. Participants were excluded if they had any other neurological diseases (apart from dementia), lacked sufficient psychological well-being to participate in the study or it was unclear whether they could effectively consent (e.g. because of the stage of their dementia or their language skills).

A total of *n* = 134 participants took part in the study, falling into three groups: (a) individuals with dementia themselves and family caregivers (spouses and other relatives) (*n* = 50); (b) professional caregivers such as employees in in-patient and out-patient care facilities, and dementia care assistants (*n* = 42); and (c) care coordinators, i.e. people not directly involved in daily caregiving activities so that they see patients with dementia less frequently, for example therapists, dementia care counsellors and physicians (*n* = 42).

The authors assert that all procedures contributing to this work comply with the ethical standards of the relevant national and institutional committees on human experimentation and with the Helsinki Declaration of 1975, as revised in 2013. All procedures involving human subjects/patients were approved by the Ethics Committee of University Medicine Greifswald (BB 024/21). Written informed consent was obtained from all participants before participation.

### Structured interview

Because the nationwide recruitment approach aimed to obtain a comprehensive overview of NPIs in dementia care across the country, interviews were mostly conducted by telephone. However, owing to participants’ preferences and logistical feasibility, six interviews were conducted in person. The interviews lasted approximately 90 min and were conducted by one of the authors; all interviewers were female and trained in communicating with people with dementia. At the beginning of the interview, the participants’ demographic data were requested. Then, structured questions were asked to determine the use and perceptions of NPIs across various domains (see below). At the end of the interview, participants were asked about perceived barriers (accessibility/no time/no money/organisational effort/personal attitude/intervention unknown/content unknown/benefits unknown/feeling insecure among strangers/stigma) to implementing NPIs.

#### Non-pharmacological interventions (NPIs)

Interviewers asked each participant ‘Which of the following therapies do you use?’, and then slowly read out the following list of NPIs: cognitive stimulation, cognitive training, massages, aromatherapy, snoezelen, phototherapy, neurofeedback, animal-assisted therapy, music therapy, art therapy, dance therapy, drama therapy, physical activity programmes and occupational therapy. After naming each NPI, the interviewer gave enough time for the participant to respond yes or no, before naming the next NPI. The list of NPIs was based on the German guidelines from 2016,^19^ scientific literature and programmes known to us. A sum score was created for each participant by counting the number of interventions the participant used.

#### Frequency of intervention use

Participants were asked how often the NPIs were being implemented. We categorised the responses as: daily (scoring 1), weekly (scoring 2) and less frequently (scoring 3). An average score was created for each participant by adding up the responses and dividing by the number of interventions the participant reported a frequency for.

#### Funding of interventions

Funding source was assessed by asking participants who financed the intervention. We categorised responses as: financed privately (scoring 1), financed through service providers (e.g. nursing home/day care centre/associations; scoring 2), prescribed by a general practitioner/neurologist (scoring 3), paid for by insurance (e.g. health/nursing insurance; scoring 4). Percentage scores were created for each participant indicating the number of interventions that the participant reported were funded by each funding source.

#### Perceived effects

Participants were asked open-ended questions about the perceived effects of the NPIs they used. To categorise the reported effects, two authors read through the answers independently. A strong congruence between the raters was reached (82.4%). Discrepancies were discussed with a third rater until consensus was reached. The final categories can be found in Supplementary Table S.1, available online at https://doi.org/10.1192/bjb.2025.10120. The percentage of NPIs for which the participant perceived effects was calculated.

### Data analysis

Complete information on the implementation of NPIs was provided by a total of 133 participants. Besides descriptive data analysis, differences were estimated via the chi-squared (*χ* ^2^) test for categorical variables, Kruskal–Wallis test in combination with Dunn’s test for pairwise continuous variables across groups, and Spearman’s rank correlation for continuous variables. For these analyses, the scores on average frequency and the percentages of funding sources and effects were log transformed.

All analyses were carried out using Stata version 16 (for Windows 10) and a significance level of *P* = 0.05.

## Results

Characteristics of the participants are shown in Supplementary Table S.2. The majority of participants were female (74.6%) and the mean age was 55.2 years (range 26–85 years). All participants had more than 8 years of education, with 58.9% having a college or university degree. About one-third in each stakeholder group perceived financial aspects, organisational effort and poor accessibility as barriers to using NPIs (other barriers were reported by fewer participants).

### Use of NPIs

Table 1 shows the rates of use of NPIs by the stakeholder groups. The NPIs that were most frequently used were cognitive stimulation (used by 87.9% or participants), cognitive training (76.7%), physical activity programmes (69.9%) and occupational therapy (61.7%). Neurofeedback and drama therapy were rarely used (<6%), and sensory interventions such as phototherapy, snoezelen, dance therapy and aromatherapy were used by fewer than 30% of participants. The percentage of participants who reported using an NPI was usually lower among people with dementia and family caregivers than among professional caregivers and care coordinators, except for cognitive training and cognitive stimulation.

**Table 1:** Differences in the use of non-pharmacological interventions (NPIs) by stakeholder group

NPI	Participants using the NPI, *n* (%)				*χ* ^2^	*P*
	Total (*n* = 133)	People with dementia and family caregivers (*n* = 49)	Professional caregivers (*n* = 42)	Care coordinators (*n* = 42)		
Cognitive stimulation	117 (87.9)	40 (81.6)	41 (97.6)	36 (85.7)	5.76	0.056
Cognitive training	102 (76.7)	32 (65.3)	34 (80.9)	36 (85.7)	5.89	0.053
Physical activity programmes	93 (69.9)	20 (40.8)	36 (85.7)	37 (88.1)	31.32	<0.001
Occupational therapy	82 (61.7)	17 (34.7)	29 (69.1)	36 (85.7)	26.32	<0.001
Massages	77 (57.9)	16 (32.7)	31 (73.8)	30 (71.4)	20.33	<0.001
Animal-assisted therapy	56 (42.1)	10 (20.4)	24 (57.1)	22 (52.4)	15.18	0.001
Music therapy	41 (30.8)	*n* < 5^a^	17 (40.5)	21 (50.0)	23.1	<0.001
Aromatherapy	37 (27.8)	*n* < 5	16 (38.1)	17 (40.5)	n.a.	n.a.
Art therapy	33 (24.8)	*n* < 5	13 (30.9)	19 (45.2)	23.86	<0.001
Dance therapy	26 (19.6)	*n* < 5	9 (21.4)	15 (35.7)	14.53	0.001
Snoezelen	24 (18.1)	*n* < 5	11 (26.2)	11 (26.2)	n.a.	n.a.
Phototherapy	17 (12.8)	*n* < 5	5 (11.9)	9 (21.4)	n.a.	n.a.
Drama therapy	7 (5.3)	*n* < 5	*n* < 5	5 (11.9)	n.a.	n.a.
Neurofeedback	5 (3.8)	*n* < 5	*n* < 5	*n* < 5	n.a.	n.a.

n.a., not applicable.

a. For subcategories with fewer than five participants no statistical analysis was carried out.

On average, stakeholders used 5.4 (s.d. = 3.0) NPIs. Significant group differences (*χ* ^2^ = 50.16, *P* < 0.001) indicated that people with dementia and their caregivers (mean 3.1, s.d. = 1.7) reported using significantly fewer NPIs than both professional caregivers (mean 6.5, s.d. = 2.5; *P* < 0.001) and care coordinators (mean 7.0, s.d. = 3.2; *P* < 0.001).

### Frequency

The average frequency of NPI use was 1.9 (s.d. = 0.4), indicating weekly implementation (only 14.1% of the participants’ averages fell into the ‘daily’ and 7.1% into the ‘less frequently’ range). There were no significant differences between the groups (*χ*² = 1.704, *P* = 0.427).

Regarding each NPI separately, only phototherapy was implemented daily; for all other NPIs, the majority of participants reported weekly implementation (Supplementary Table S.3).

### Funding

Of all NPIs, on average, 24.5% (s.d. = 38.1%) were funded by an external service provider such as a nursing home or day care facility, 22.6% (s.d. = 34.1%) were financed privately, 25.9% (s.d. = 37.2%) were financed through prescriptions and 17.4% (s.d. = 33.3%) were covered by insurance benefits. There was no statistically significant difference between the stakeholder groups in the percentage of NPIs financed privately (*χ*² = 5.590, *P* = 0.061) or provided as insurance benefits (*χ*² = 0.849, *P* = 0.654). People with dementia and family caregivers reported a significantly lower percentage of NPIs funded by service providers (10.8%, s.d. = 23.5%, *χ*² = 9.878, *P* = 0.007) than the percentages reported by the care coordinators (17.9%, s.d. = 36.2%) and professional caregivers (47.5%, s.d. = 43.9%; Dunn’s test: professional caregivers versus people with dementia and family caregivers *P* < 0.001, care coordinators versus people with dementia and family caregivers *P* = 0.011, care coordinators versus professional caregivers *P* = 0.338). Moreover, the percentage of NPIs funded by prescriptions was significantly lower among professional caregivers (11.8%, s.d. = 20.9%, *χ*² = 15.250, *P* < 0.001) than among people with dementia and family caregivers (28.3%, s.d. = 37.5%) and care coordinators (37.3%, s.d. = 45.1%) (Dunn’s test: professional caregivers versus people with dementia and family caregivers *P* < 0.001, professional caregivers versus care coordinators *P* < 0.001, care coordinators versus people with dementia and family caregivers *P* = 0.227).

**Fig. 1:**
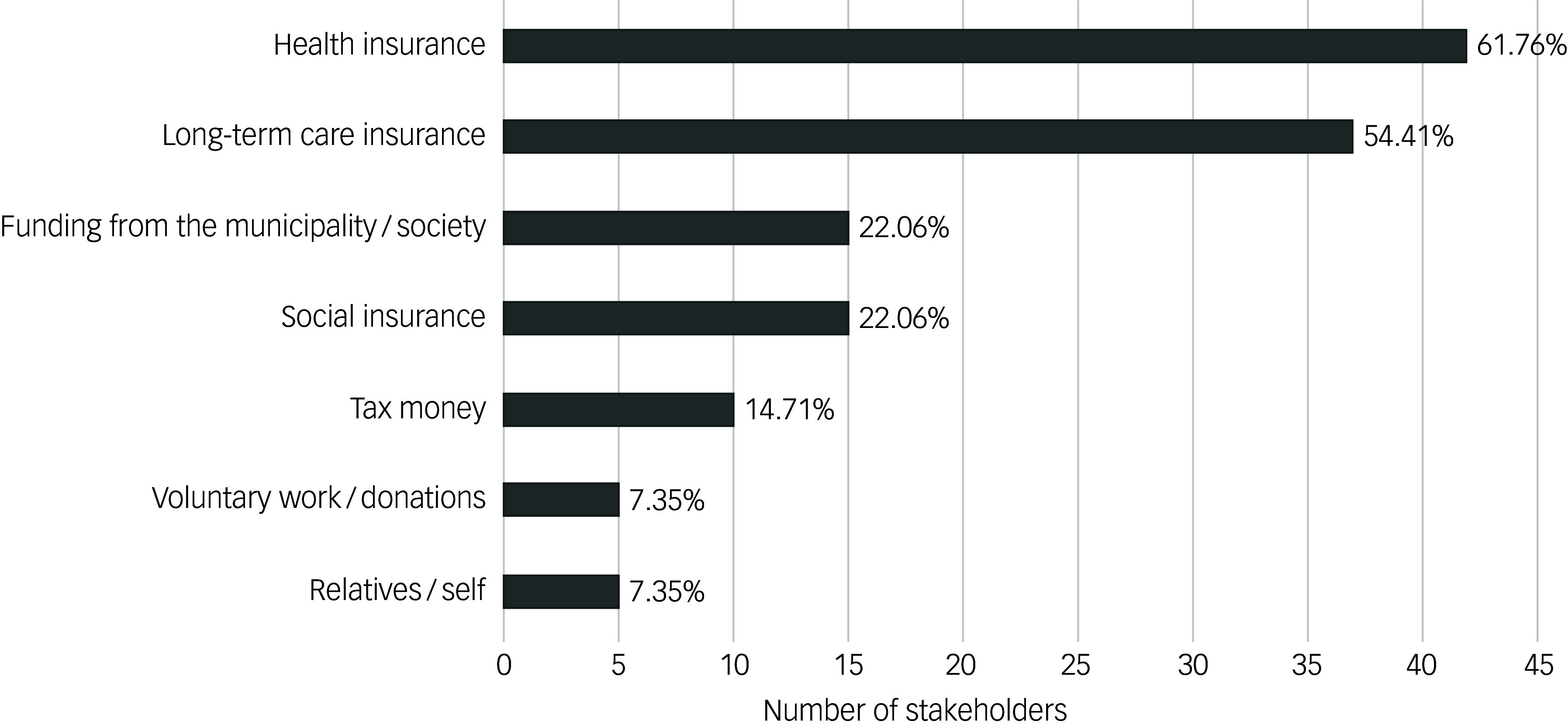
Study participants’ suggestions on how non-pharmacological interventions in dementia care should be funded.

When asked how NPIs in dementia care should be funded, 57.6% of participants expressed doubt that everyone can afford them. Fig. 1 shows the funding sources that participants suggested to cover the costs of NPIs. More than 60% suggested that health insurance should cover the costs and more than half specifically suggested long-term care insurance. Only a small percentage (∼7%) of the participants suggested that costs should be covered privately or should come in the form of voluntary work or donations.

### Perceived effects

For the NPIs that participants used, on average, they perceived effects for 90.3% (s.d. = 31.3%). Even though this average was lower among people with dementia and family caregivers (79.9%, s.d. = 28.3%) compared with professional caregivers (97.9%, s.d. = 43.6%) and care coordinators (94.6%, s.d. = 10.1%), the difference was not statistically significant (*χ*² = 4.140, *P* = 0.126).

Regarding each NPI separately, as displayed in Table 2, more than 80% of participants reported perceiving effects of the NPIs they used. The highest percentages of participants reported perceived effects for phototherapy, dance therapy and physical activity programmes and the lowest percentages reported effects for occupational therapy. Improved well-being was the most commonly reported effect and most frequently reported to be achieved through art and drama therapy. Activation was most commonly perceived to be achieved through cognitive stimulation, relaxation through massage, and maintaining abilities through occupational therapy.

**Table 2:** Participants’ (*n* = 133) reports of perceived effects of non-pharmacological interventions (NPIs)

NPI	Any effect, *n* (%)	Activation, *n* (%)	Appreciation, *n* (%)	Promotion of well-being, *n* (%)	Promotion of social health, *n* (%)	Maintaining abilities, *n* (%)	Relaxation, *n* (%)	Improve cognition, *n* (%)	Improve motor skills, *n* (%)	Reduction of behavioural problems, *n* (%)
Cognitive stimulation	111 (95.7)	57 (48.7)	20 (17.1)	64 (54.7)	12 (10.3)	8 (6.8)	7 (5.9)	–	–	–
Cognitive training	87 (85.3)	16 (15.7)	6 (5.9)	63 (61.8)	–	15 (14.7)	9 (8.8)	24 (23.5)	–	–
Massages	74 (96.1)	7 (9.1)	–	30 (38.9)	20 (25.9)	–	51 (66.2)	–	–	5 (6.5)
Aromatherapy	33 (89.2)	12 (32.4)	–	11 (29.7)	–	–	21 (56.8)	–	–	–
Snoezelen	21 (87.5)	6 (25.0)	–	–	–	–	15 (62.5)	–	–	–
Phototherapy	17 (100.0)	6 (35.3)	–	7 (41.2)	–	–	–	–	–	–
Neurofeedback	–	–	–	–	–	–	–	–	–	–
Animal-assisted therapy	53 (94.6)	21 (37.5)	–	36 (64.3)	8 (14.3)	–	16 (28.6)	–	–	–
Music therapy	39 (95.1)	12 (29.3)	–	31 (75.6)	–	–	–	–	–	–
Art therapy	31 (93.9)	5 (15.2)	–	28 (84.9)	–	–	–	–	–	–
Dance therapy	26 (100.0)	9 (34.6)	–	20 (76.9)	–	–	–	–	–	–
Drama therapy	6 (85.7)	–	–	6 (85.7)	–	–	–	–	–	–
Physical activity programmes	91 (97.9)	24 (25.8)	–	48 (51.6)	10 (10.8)	24 (25.8)	12 (12.9)	–	10 (10.8)	–
Occupational therapy	66 (80.5)	10 (12.2)	–	26 (31.7)	–	37 (45.1)	–	–	–	–

### Associations with NPI use

Associations between participants’ characteristics and perceptions and the number of NPIs used are shown in Table 3. Among people with dementia and family caregivers, only a greater percentage of NPIs funded privately or by insurance was associated with a lower number of NPIs used. Similarly, among professional caregivers, a greater percentage of NPIs funded through prescriptions was associated with a lower number of NPIs used. This indicates that funding sources might limit the possibility of implementing NPIs. Among care coordinators, a greater percentage of NPIs funded through insurance as well as a higher frequency of using NPIs were associated with a lower number of NPIs used.

**Table 3:** Associations between participants’ (*n* = 133) characteristics and perceptions and the number of non-pharmacological therapeutic interventions (NPIs) used

	Number of NPIs used								
	People with dementia and family caregivers			Professional caregivers			Care coordinators		
		*r*	*P*		*r*	*P*		*r*	*P*
Age		0.106	0.469		−0.145	0.360		−0.078	0.623
Frequency of NPI use, mean^a^		−0.207	0.206		−0.167	0.329		−0.463	0.023
Effects perceived, %^a^		−0.070	0.648		−0.114	0.478		0.002	0.988
Privately funded, %^a^		−0.446	0.017		−0.367	0.331		−0.029	0.922
Service-provider funded, %^a^		0.078	0.819		0.034	0.874		−0.605	0.064
Prescription funded, %^a^		−0.229	0.305		−0.617	0.011		−0.381	0.088
Insurance funded, %^a^		−0.759	0.029		−0.431	0.109		−0.549	0.042

	Mean (s.d.)	*χ*²	*P*	Mean (s.d.)	*χ*²	*P*	Mean (s.d.)	*χ*²	*P*
Gender									
Female	3.1 (1.9)	0.113	0.737	6.2 (2.2)	0.721	0.396	6.9 (3.1)	0.352	0.553
Male	3.1 (1.3)			7.0 (3.0)			7.8 (3.9)		
Education									
Low	2.8 (1.6)	1.085	0.298	6.6 (1.9)	0.166	0.684	6.1 (2.7)	2.141	0.143
High	3.3 (1.8)			6.4 (2.7)			7.6 (3.3)		
Barriers									
Poor accessibility									
No	2.9 (1.8)	1.130	0.288	6.4 (2.5)	0.016	0.898	7.2 (3.0)	0.365	0.546
Yes	3.4 (1.5)			6.8 (2.1)			6.7 (3.5)		
No time									
No	3.0 (1.8)	1.760	0.185	6.2 (2.5)	1.405	0.236	6.8 (3.2)	0.611	0.434
Yes	3.7 (1.0)			7.4 (2.1)			7.9 (3.2)		
Financial aspects									
No	2.7 (1.4)	3.374	0.066	6.2 (2.5)	0.847	0.357	6.5 (3.2)	1.451	0.228
Yes	3.9 (2.2)			7.0 (2.4)			7.8 (3.1)		
Organisational effort									
No	3.2 (1.8)	0.438	0.508	6.2 (2.5)	1.119	0.290	7.0 (3.1)	0.003	0.956
Yes	2.8 (1.5)			7.0 (2.3)			7.1 (3.6)		
Personal attitude									
No	3.1 (1.7)	0.005	0.946	6.7 (2.5)	3.295	0.069	6.8 (3.3)	1.338	0.247
Yes	3.1 (2.0)			5.0 (1.3)			9.0 (0.8)		
Intervention unknown									
No	3.1 (1.8)	0.022	0.882	6.3 (2.4)	0.500	0.479	6.9 (3.1)	0.370	0.543
Yes	3.0 (1.2)			7.5 (3.1)			7.6 (3.8)		
Benefits unknown									
No	3.1 (1.9)	0.542	0.462	6.2 (2.4)	3.718	0.054	7.4 (3.3)	0.868	0.351
Yes	3.2 (0.9)			8.5 (1.9)			6.3 (2.9)		

a. log transformed.

## Discussion

With this study, we wanted to understand better if and what NPIs are used in dementia care. Cognitive training and stimulation, physical activity programmes and occupational therapy seem the most frequently used NPIs. In contrast, snoezelen, aromatherapy, dance therapy, neurofeedback and drama therapy were rarely implemented, despite the fact that, for instance, snoezelen^20^ and dance therapy^21^ are explicitly recommended in systematic reviews. Implementation frequency was mainly weekly. People with dementia and family caregivers reported using significantly fewer NPIs (except cognitive training/stimulation) than professional caregivers and care coordinators, which might have resulted in the fact that they had a tendency to perceive fewer effects. Yet, family caregivers may also just be too close to notice significant improvements.^22,23^ Nonetheless, they did perceive some positive effects, suggesting that using fewer NPIs is not the result of a lack of perceived efficacy. Previous studies reported that people with dementia and their caregivers perceive positive changes in BPSD and increased well-being and quality of life due to non-pharmacological therapies, such as exercise programmes and occupational therapy.^24,25^ Rather, a lower utilisation in this group could be attributed to the factor that family members may not know about them or face constraints that limit their capacity to get involved in too many interventions. High levels of stress and burnout^26,27^ among informal caregivers could lead to the decision to implement fewer NPIs. Accordingly, people with dementia living at home might have more difficulties accessing therapeutic interventions than those living in nursing homes, where therapies are offered on-site. In contrast, professional caregivers and care coordinators typically have better access to information, resources and infrastructure that facilitate the implementation of a broader range of NPIs. This shows in higher utilisation rates.

Participants reported perceiving effects for all NPIs. This is consistent with scientific findings, which show that music therapy as well as cognitive and sensory interventions enhance well-being; exercise programmes, cognitive interventions and aromatherapy activate people with dementia; and animal-assisted therapy and sensory measures provide relaxation.^15,28^ Owing to the small number of people that use snoezelen, aromatherapy, dance therapy and drama therapy, less information regarding real-life efficacy is available. However, a similar proportion of participants (>85%) reported perceiving effects for those NPIs as for the most frequently used NPIs (cognitive stimulation and cognitive training). Lower rates of use might simply be the result of a lack of availability. Further research is needed to determine that.

Funding of NPIs seems to come from several sources: private (mostly cognitive stimulation and music therapy), service providers themselves (e.g. snoezelen, aromatherapy, phototherapy, animal-assisted therapy) and insurance (mainly occupational therapy). People with dementia and family caregivers used more prescribed interventions, whereas professional caregivers and care coordinators reported more interventions funded by service providers. These differences may reflect differences in professional knowledge and access to funding information. A lack of knowledge among informal caregivers has been noted in several studies, which subsequently call for better knowledge distribution.^29,30^ A recently published study revealed that providing support in primary care – for instance, in the form of training and interdisciplinary team work – can improve the quality of dementia care.^31^ A lack of knowledge can be a barrier that people themselves are not necessarily aware of, as this information is just not part of their expectation. Interestingly, in our study, funding sources determined the number of NPIs used. Implementing more interventions comes with more costs overall, so that people may restrict their use to avoid financial strain. Our participants doubted that the necessary costs of interventions can be covered by individuals and their families alone. This was also observed in other studies, in which both health professionals and informal caregivers mentioned insufficient funds and inflexible service conditions as barriers for NPI use.^32–34^ Accordingly, it is crucial to obtain alternative funding sources. The investment would pay off in the long term, as a systematic review found that cognitive stimulation, tailored activity programmes and occupational therapy are more cost-effective than usual care.^35^

### Limitations

A limitation of the current study is that, although we aimed to reach as many people involved in dementia care as possible, those who participated may not be representative of everyone. The decision not to participate in our interviews may also reflect a lack of time for or interest in NPIs themselves. Accordingly, our findings may overestimate the true implementation rate. Moreover, we did not offer the interview in languages other than German, so that the experiences of recently migrated individuals could not be considered. Hence, the results can only serve as an orientation and cannot be generalised. They reflect the subjective perceptions of the participants, and objective parameters may provide a different picture. A few participants (*n* = 6) provided their answers in face-to-face interviews, and we cannot exclude the possibility that aspects of social interaction (e.g. facial expression, gestures) may have affected their answers. Moreover, use of NPIs may depend on the neuropsychiatric symptoms that people with dementia have and these symptoms differ between patients. For clinical practice, it would be valuable to know for what specific symptoms each NPI is used and whether it is perceived to be effective. The effects that the participants reported may depend on that as well as on the specific components of the individual NPI. Further, as this work is part of a larger study, we cannot differentiate whether the barriers mentioned are specifically related to NPIs or to non-pharmacological aspects of dementia care in general.

### Implications

Our findings that some NPIs are much more frequently implemented than others in dementia care and that people with dementia and family caregivers reported less frequent use than professionals may reflect differences in knowledge and as yet unexplored barriers to use. Future research should identify targeted strategies to promote the use of NPIs, ensuring that effective interventions can be successfully integrated into both home-based and institutional care settings.

## Supporting information

Ross et al. supplementary material Ross et al. supplementary material

## Data Availability

The data are not publicly available owing to legal restrictions. Eligible requests can be made to the corresponding author, F.S.R.

## About the authors

**Sabrina D. Ross**, PhD, is a researcher in the German Center for Neurodegenerative Diseases (DZNE), Research Group (RG) Psychosocial Epidemiology & Public Health, Greifswald, Germany. **Nadja Ziegert**, BSc, is a study manager in the German Center for Neurodegenerative Diseases (DZNE), RG Psychosocial Epidemiology & Public Health, Greifswald, Germany. **Francisca S. Rodriguez**, PhD, is research group head in the German Center for Neurodegenerative Diseases (DZNE), RG Psychosocial Epidemiology & Public Health, Greifswald, Germany.

## References

[1] National Research Council. Aging and the Macroeconomy: Long-Term Implications of an Older Population. National Academies Press, 2013. 23885367

[2] Shin JH. Dementia epidemiology fact sheet 2022. Ann Rehabil Med 2022; 46: 53–9. 35508924 10.5535/arm.22027 PMC9081392

[3] World Health Organization. Global Status Report on the Public Health Response to Dementia. WHO, 2021.

[4] Sheehan OC, Haley WE, Howard VJ, Huang J, Rhodes JD, Roth DL. Stress, burden, and well-being in dementia and nondementia caregivers: insights from the caregiving transitions study. Gerontologist 2021; 61: 670–9. 32816014 10.1093/geront/gnaa108 PMC8276607

[5] De Deyn PP, Katz IR, Brodaty H, Lyons B, Greenspan A, Burns A. Management of agitation, aggression, and psychosis associated with dementia: a pooled analysis including three randomized, placebo-controlled double-blind trials in nursing home residents treated with risperidone. Clin Neurol Neurosurg 2005; 107: 497–508. 15922506 10.1016/j.clineuro.2005.03.013

[6] Feast A, Orrell M, Charlesworth G, Melunsky N, Poland F, Moniz-Cook E. Behavioural and psychological symptoms in dementia and the challenges for family carers: systematic review. Br J Psychiatry 2016; 208: 429–34. 26989095 10.1192/bjp.bp.114.153684 PMC4853642

[7] McDermott O, Charlesworth G, Hogervorst E, Stoner C, Moniz-Cook E, Spector A, et al. Psychosocial interventions for people with dementia: a synthesis of systematic reviews. Aging Ment Health 2019; 23: 393–403. 29338323 10.1080/13607863.2017.1423031

[8] Chalfont G, Milligan C, Simpson J. A mixed methods systematic review of multimodal non-pharmacological interventions to improve cognition for people with dementia. Dementia (London) 2020; 19: 1086–130. 30193536 10.1177/1471301218795289 PMC7180318

[9] Woods B, Aguirre E, Spector AE, Orrell M. Cognitive stimulation to improve cognitive functioning in people with dementia. Cochrane Database Syst Rev 2012; 2: CD005562. 10.1002/14651858.CD005562.pub2 22336813

[10] Ball EL, Owen-Booth B, Gray A, Shenkin SD, Hewitt J, McCleery J. Aromatherapy for dementia. Cochrane Database Syst Rev 2020; 8: CD003150. 32813272 10.1002/14651858.CD003150.pub3 PMC7437395

[11] Baek SM, Lee Y, Sohng KY. The psychological and behavioural effects of an animal-assisted therapy programme in Korean older adults with dementia. Psychogeriatrics 2020; 20: 645–53. 32291838 10.1111/psyg.12554 PMC7586947

[12] Moreno-Morales C, Calero R, Moreno-Morales P, Pintado C. Music therapy in the treatment of dementia: a systematic review and meta-analysis. Front Med 2020; 19: 160. 10.3389/fmed.2020.00160 PMC7248378 32509790

[13] Chowdhary N, Barbui C, Anstey KJ, Kivipelto M, Barbera M, Peters R, et al. Reducing the risk of cognitive decline and dementia: WHO recommendations. Front Neurol 2021; 12: 765584. 35082745 10.3389/fneur.2021.765584 PMC8784726

[14] de Oliveira AM, Radanovic M, de Mello PC, Buchain PC, Vizzotto AD, Celestino DL, et al. Nonpharmacological interventions to reduce behavioral and psychological symptoms of dementia: a systematic review. Biomed Res Int 2015; 2015: 218980. 26693477 10.1155/2015/218980 PMC4676992

[15] Meyer C, O’Keefe F. Non-pharmacological interventions for people with dementia: a review of reviews. Dementia (London) 2020; 19: 1927–54. 30526036 10.1177/1471301218813234

[16] Fossey J, Garrod L, Tolbol Froiland C, Ballard C, Lawrence V, Testad I. What influences the sustainability of an effective psychosocial intervention for people with dementia living in care homes? A 9 to 12-month follow-up of the perceptions of staff in care homes involved in the WHELD randomised controlled trail. Int J Geriatr Psychiatry 2019; 34: 674–82. 30706523 10.1002/gps.5066 PMC6594193

[17] Park J, Howard H, Tolea MI, Galvin JE. Perceived benefits of using nonpharmacological interventions in older adults with Alzheimer’s disease or dementia with Lewy bodies. J Gerontol Nurs 2020; 46: 37–46. 10.3928/00989134-20191217-01 31895960

[18] Bennett S, Laver K, MacAndrew M, Beattie E, Clemson L, Runge C, et al. Implementation of evidence-based, non-pharmacological interventions addressing behavior and psychological symptoms of dementia: a systematic review focused on implementation strategies. Int Psychogeriatr 2021; 33: 947–75. 33190660 10.1017/S1041610220001702

[19] Deutsche Gesellschaft für Psychiatrie und Psychotherapie, Psychosomatik und Nervenheilkunde, Deutsche Gesellschaft für Neurologie. S3-Leitlinie “Demenzen” [S3 Guideline on Dementia]. Deutsche Gesellschaft für Neurologie, 2016.

[20] Pinto JO, Dores AR, Geraldo A, Peixoto B, Barbosa F. Sensory stimulation programs in dementia: a systematic review of methods and effectiveness. Expert Rev Neurother 2020; 20: 1229–47. 32940543 10.1080/14737175.2020.1825942

[21] Salihu D, Wong EML, Bello UM, Kwan RYC. Effects of dance intervention on agitation and cognitive functioning of people living with dementia in institutional care facilities: systematic review. Geriatr Nurs 2021; 42: 1332–40. 34560528 10.1016/j.gerinurse.2021.08.015

[22] Neumann PJ, Araki SS, Gutterman EM. The use of proxy respondents in studies of older adults: lessons, challenges, and opportunities. J Am Geriatr Soc 2000; 48: 1646–54. 11129756 10.1111/j.1532-5415.2000.tb03877.x

[23] Lopez A, Tinella L, Caffo A, Bosco A. Measuring the reliability of proxy respondents in behavioural assessments: an open question. Aging Clin Exp Res 2023; 35: 2173–90. 37540380 10.1007/s40520-023-02501-z PMC10520105

[24] Patel B, Perera M, Pendleton J, Richman A, Majumdar B. Psychosocial interventions for dementia: from evidence to practice. Adv Psychiatr Treatm 2014; 20: 340–9.

[25] Hulme C, Wright J, Crocker T, Oluboyede Y, House A. Non-pharmacological approaches for dementia that informal carers might try or access: a systematic review. Int J Geriatr Psychiatry 2010; 25: 756–63. 19946870 10.1002/gps.2429

[26] Gérain P, Zech E. Do informal caregivers experience more burnout? A meta-analytic study. Psychol Health Med 2021; 26: 145–61. 32816530 10.1080/13548506.2020.1803372

[27] Alves LCS, Monteiro DQ, Bento SR, Hayashi VD, Pelegrini LNC, Vale FAC. Burnout syndrome in informal caregivers of older adults with dementia: a systematic review. Dement Neuropsychol 2019; 13: 415–21. 31844495 10.1590/1980-57642018dn13-040008 PMC6907708

[28] Scales K, Zimmerman S, Miller SJ. Evidence-based nonpharmacological practices to address behavioral and psychological symptoms of dementia. Gerontologist 2018; 58(suppl 1): s88–102. 29361069 10.1093/geront/gnx167 PMC5881760

[29] Britton A, Zimmermann M. Informal dementia care: the carer’s lived experience at the divides between policy and practice. Dementia (London) 2022; 21: 2117–27. 35838118 10.1177/14713012221112234 PMC9483676

[30] Lindeza P, Rodrigues M, Costa J, Guerreiro M, Rosa MM. Impact of dementia on informal care: a systematic review of family caregivers’ perceptions. BMJ Support Palliat Care 2020; 14: e38–49. 10.1136/bmjspcare-2020-002242 33055092

[31] Henein M, Arsenault-Lapierre G, Sourial N, Godard-Sebillotte C, Vedel I. The association between the level of institutional support for dementia care in primary care practices and the quality of dementia primary care: A retrospective chart review. Alzheimers Dement (N Y) 2022; 8: e12233. 35128028 10.1002/trc2.12233 PMC8804918

[32] Ross SD, Ziegert N, Rodriguez FS. Identifying relevant psychosocial factors in the care of people with dementia: findings of a focus group study with health professionals and informal caregivers. J Public Health (Berl) 2025; 33: 1271–80.

[33] Cohen-Mansfield J, Thein K, Marx MS, Dakheel-Ali M. What are the barriers to performing nonpharmacological interventions for behavioral symptoms in the nursing home? J Am Med Dir Assoc 2012; 13: 400–5. 21872537 10.1016/j.jamda.2011.07.006 PMC3262905

[34] Md Hussin NS, Karuppannan M, Gopalan Y, Tan KM, Gnanasan S. Exploration of non-pharmacological interventions in the management of behavioural and psychological symptoms of dementia. Singapore Med J 2023; 64: 497–502. 34600449 10.11622/smedj.2021125 PMC10476919

[35] Knapp M, Iemmi V, Romeo R. Dementia care costs and outcomes: a systematic review. Int J Geriatr Psychiatry 2013; 28: 551–61. 22887331 10.1002/gps.3864

