# Supplementary material for: Non-pharmacological interventions in dementia care: what is being implemented
Source: BJPsych Bull. 2025 Jul 7;50(2):138–45. doi: 10.1192/bjb.2025.10120 (PMC13150545; doi:10.1192/bjb.2025.10120)
Supplement: Ross et al. supplementary material [file S2056469425101204sup001.pdf]

Supplementary material

To the manuscript "Non-pharmacological therapeutic interventions in dementia care"

Table S.1. Definitions and examples of the categorized effects.

Effect	Definition	Example
Activation	Stimulation of physical, cognitive, motor, and sensory domains	- Cognition is promoted/stimulated<br>- Conversation-stimulating / narrative-stimulating<br>- Stimulating activity<br>- Awakening memories<br>- Stimulating the senses<br>- Stimulating movement<br>- Stimulate the imagination<br>- Get new impulses/impressions; discover new things<br>- Creating new stimuli; getting 'input from outside'<br>- Promotion of attention/concentration<br>- Increasing body awareness
Appreciation	Experience of external recognition and appreciation from other people	- Feeling valued<br>- Being praised; receiving confirmation from outside/by other people<br>- Feeling seen/heard; feeling accepted, feeling taken seriously, feeling noticed<br>- Recognition of the situation/the biography/the person/the interests<br>- Promoting identity
Improved well-being	Self-perceived positive feeling of physical and/or mental well-being	- Have success (experiences)<br>- (Self) experience of own competencies/skills, independence, performance, feeling "I can still do something"/"I can do more than I thought I could"<br>- (Self) experience of self-worth (feeling), self-efficacy,<br>- Joy, fun<br>- Perception of quality of life<br>- Maintaining mental well-being<br>- Confidence<br>- Mood-enhancing<br>- Be proud<br>- Feeling 'needed'
Improved social health	Interacting and coming into contact with other people	- Being in exchange/interaction with other people<br>- Having conversations, being approached<br>- Doing or experiencing something together with other people<br>- Having social contacts/being "networked"<br>- Experience social participation/inclusion in a social community/feeling of belonging to a group<br>- Avoiding isolation and loneliness, "I don't feel alone"<br>- Being in (trusting) relationships with other people/interacting with other people<br>- Strengthening social relationships<br>- Having trust in other people/ being able to confide in other people<br>- Experience closeness and affection from other people
Maintaining abilities	Cognitive/motor skills are maintained/improved in order to maintain independence	- Maintaining skills/movement/motor skills/independence
Relaxation	A state in which body, mind and soul are in harmony with each other	- Soothing<br>- Calmer<br>- relaxing
Improved cognition	Improvement of a cognitive domain	- Addressing long-term memory<br>- Improved ability to concentrate<br>- Better memory<br>- Cognitive abilities return
Improved motor skills	Improvement of a motor domain	- Better agility<br>- Improved mobility
Reduction of behavioral problems	Improvement of behavioral problems	- Behavioral problems are reduced<br>- Promotion of day/night rhythm

Table S.2. Characteristics of the study participants (n=133).

		**People with dementia and family caregivers (n=50)**	**Professional caregivers (n=42)**	**Care coordinators (n=42)**
		**Mean, SD**	**Mean, SD**	**Mean, SD**
**Age**	Age	64.5 years, 11.7 years	51.1 years, 12.9 years	48.3 years, 12.6 years
		**Frequency (n, %)**	**Frequency (n, %)**	**Frequency (n, %)**
**Gender**	Female	34 (68.0 %)	30 (71.4 %)	36 (85.7 %)
	Male	16 (32.0 %)	12 (28.6 %)	6 (14.3 %)
**Education** §	Low	24 (43.6 %)	15 (27.3 %)	16 (38.1 %)
	High	26 (32.9 %)	27 (34.2 %)	26 (32.9 %)
**Barriers**	Financial aspects	14 (28.0 %)	14 (33.3 %)	16 (38.1 %)
	Organizational effort	17 (34.0 %)	12 (28.6 %)	12 (28.6 %)
	Poor accessibility	15 (30.0 %)	8 (19.1 %)	15 (35.7 %)
	No time	6 (12.0%)	8 (19.1%)	9 (21.4%)
	Personal attitude	8 (16.0%)	6 (14.3%)	n<5
	Unknown intervention	5 (10.0%)	n<5	8 (19.1%)
	Benefits unknown	9 (18.0%)	n<5	15 (35.7%)

Notes:§ = High education are those who completed college or university and low education is those who did not; n = Number of participants in the corresponding group; % = Frequency in the group in per cent; SD = Standard Deviation

Table S.3. Reported frequency of non-pharmacological therapeutic intervention use (NPTI, n=133).

	**Daily (N, %)**				**Weekly (N, %)**				**Less frequently (N, %)**			
	**Total**	**People with dementia and family caregivers**	**Professional caregivers**	**Care coordinators**	**Total**	**People with dementia and family caregivers**	**Professional caregivers**	**Care coordinators**	**Total**	**People with dementia and family caregivers**	**Profession al caregivers**	**Care coordinato rs**
Cognitive stimulation	26 (42.6 %)	7 (43.8 %)	13 (48.2 %)	6 (33.3 %)	32 (52.5 %)	7 (43.8 %)	14 (51.9 %)	11 (61.1 %)	n<5	n<5	-	n<5
Cognitive training	28 (38.9 %)	8 (28.6 %)	15 (55.6 %)	5 (29.4 %)	43 (59.7 %)	19 (67.9 %)	12 (44.4 %)	12 (70.6 %)	n<5	n<5	-	-
Massages	n<5	-	n<5	-	19 (79.2 %)	5 (71.4 %)	6 (66.7 %)	8 (100.0 %)	n<5	n<5	n<5	-
Phototherapy	6 (100.0 %)	n<5	n<5	n<5	-	-	-	-	-	-	-	-
Animal-assisted therapy	7 (28.0 %)				12 (48.0 %)				6 (24 %)			
Music therapy	n<5	-	n<5	-	11 (84.6 %)	n<5	7 (77.8 %)	n<5	n<5	-	n<5	-
Art therapy	-	-	-	-	7 (70.0 %)	-	5 (62.5 %)	n<5	n<5	-	n<5	-
Physical activity programs	20 (31.8 %)				43 (68.3 %)				-			
Occupational therapy	-				31 (93.9 %)				n<5			

Notes: Not shown is Snoezeln, Neurofeedback, Drama therapy, Aromatherapy, and Dance therapy as there was no cell for which at least five participants reported a frequency. *Abbreviations:* n = number of participants; % = Percentage of those implementing the intervention
